# Examining the diet quality of Canadian adults and the alignment of Canadian front-of-pack labelling regulations with other front-of-pack labelling systems and dietary guidelines

**DOI:** 10.3389/fpubh.2023.1168745

**Published:** 2023-06-23

**Authors:** Jennifer J. Lee, Mavra Ahmed, Chantal Julia, Alena Praneet Ng, Laura Paper, Wendy Y. Lou, Mary R. L’Abbé

**Affiliations:** ^1^Department of Nutritional Sciences, Temerty Faculty of Medicine, University of Toronto, Toronto, ON, Canada; ^2^Joannah and Brian Lawson Centre for Child Nutrition, University of Toronto, Toronto, ON, Canada; ^3^Sorbonne Paris Nord University, INSERM, INRAE, CNAM, Nutritional Epidemiology Research Team (EREN), Epidemiology and Statistics Research Center, University of Paris (CRESS), Bobigny, France; ^4^Public Health Department, Avicenne Hospital, AP-HP, Bobigny, France; ^5^Nutritional Epidemiology Surveillance Team (ESEN), Santé Publique France, The French Public Health Agency, Bobigny, France; ^6^Biostatistics Division, Dalla Lana School of Public Health, University of Toronto, Toronto, ON, Canada

**Keywords:** front-of-pack, FOPL, dietary patterns, nutrient profiling, HEFI, Nutri-score, DASH, DCCP

## Abstract

**Introduction:**

Canada promulgated mandatory front-of-pack labelling (FOPL) regulations in 2022, requiring pre-packaged foods meeting and/or exceeding recommended thresholds for nutrients-of-concern (i.e., saturated fat, sodium, sugars) to display a “high-in” nutrition symbol. However, there is limited evidence on how Canadian FOPL (CAN-FOPL) regulations compare to other FOPL systems and dietary guidelines. Therefore, the objectives of the study were to examine the diet quality of Canadians using the CAN-FOPL dietary index system and its alignment with other FOPL systems and dietary guidelines.

**Methods:**

Nationally representative dietary data from the 2015 Canadian Community Health Survey-Nutrition survey (*n* = 13,495) was assigned dietary index scores that underpin CAN-FOPL, Diabetes Canada Clinical Practice (DCCP) Guidelines, Nutri-score, Dietary Approaches to Stop Hypertension (DASH) and Canada’s Food Guide (Healthy Eating Food Index-2019 [HEFI-2019]). Diet quality was examined by assessing linear trends of nutrient intakes across quintile groups of CAN-FOPL dietary index scores. The alignment of CAN-FOPL dietary index system compared with other dietary index systems, with HEFI as the reference standard, was examined using Pearson’s correlations and к statistics.

**Results:**

The mean [95% CI] dietary index scores (range: 0–100) for CAN-FOPL, DCCP, Nutri-score, DASH, and HEFI-2019 were 73.0 [72.8, 73.2], 64.2 [64.0, 64.3], 54.9 [54.7, 55.1], 51.7 [51.4, 51.9], and 54.3 [54.1, 54.6], respectively. Moving from the “least healthy” to the “most healthy” quintile in the CAN-FOPL dietary index system, intakes of protein, fiber, vitamin A, vitamin C, and potassium increased, while intakes of energy, saturated fat, total and free sugars, and sodium decreased. CAN-FOPL showed moderate association with DCCP (*r* = 0.545, *p* < 0.001), Nutri-score (*r* = 0.444, *p* < 0.001),  and HEFI-2019 (*r* = 0.401, *p* < 0.001), but poor association with DASH (*r* = 0.242, *p* < 0.001). Slight to fair agreement was seen between quintile combinations of CAN-FOPL and all dietary index scores (*к* = 0.05–0.38).

**Discussion:**

Our findings show that CAN-FOPL rates the dietary quality of Canadian adults to be healthier than other systems. The disagreement between CAN-FOPL with other systems suggest a need to provide additional guidance to help Canadians select and consume ‘healthier’ options among foods that would not display a front-of-pack nutrition symbol.

## 1. Introduction

Unhealthy diet is one of the major modifiable risk factors for non-communicable diseases (NCDs) (1). Front-of-pack labelling (FOPL) has been recognized as an effective public health strategy to target unhealth dietary patterns (2, 3), and many countries have implemented mandatory FOPL regulations (e.g., “high-in” warning labels for sugars, sodium, saturated fats, and total energy in Chile and Mexico; red warning labels for sugars, sodium, and saturated fats in Israel) or introduced a government-led voluntary program (e.g., Nutri-score in France; Healthy Star Ratings in Australia and New Zealand; green positive label in Israel) (4–6). In 2022, Canada published FOPL regulations to mandate pre-packaged foods and beverages meeting and/or exceeding thresholds for nutrients-of-concern (i.e., saturated fat, sodium, and sugars) to display a “high-in” nutrition symbol at the front of the package (7). A recent study showed that foods that would display a “high in” front-of-pack symbol, according to Canadian FOPL regulations, contribute to 15–40% of intakes of nutrients-of-public health concern among Canadian adults (8). Although one of the key guiding principles of effective FOPL is the consistency with other dietary guidelines (3), it remains unclear how Canadian FOPL regulations compare to other FOPL systems and dietary guidelines for Canadians, particularly for those with risk factors for NCDs that are more vulnerable to unhealthy diets.

Several healthy guidelines and recommendations currently exist for Canadians. Canada’s Food Guide, the latest evidence-based national dietary guidelines for all Canadians, was released in 2019 (9). Canada’s Food Guide was designed to promote healthy eating, overall nutritional well-being, and reduce risk of nutrition-related NCDs (10, 11). A dietary index scoring system based on recommendations from Canada’s Food Guide 2019, also known as the Healthy Eating Food Index (HEFI)-2019 (12, 13) showed greater adherence to Canada’s Food Guide can reduce risk of cardiovascular diseases (14). Diabetes Canada has published dietary recommendations for Canadians with or at risk for diabetes in the Diabetes Canada Clinical Practice Guidelines (DCCP) to treat and self-manage pre-diabetes and diabetes (15). While promoting individualized dietary patterns, the DCCP recommend the consumption of certain foods or nutrients (e.g., nuts, plant-based protein foods) and limiting the intakes of others (e.g., high glycemic index foods, saturated fat) (15). A nutrient profiling model, which classifies or ranks foods according to their nutritional composition for reasons related to preventing disease and promoting health (16), was recently developed to assess the alignment of foods and beverages with the DCCP (17). When converted to a dietary index system, the DCCP nutrient profiling model discriminated nutrient and food consumption in a cohort of French adults (18). Similarly, other nutrient profiling models underpinning FOPL systems have shown to be a valid tool in examining diet quality. For instance, the UK’s Food Standards Agency nutrient profiling model, which underpins many FOPL systems (e.g., Nutri-score, Ofcom) has shown positive associations with risk of cardiovascular diseases (19, 20), cancer (21, 22), and overweight and obesity (23). In addition to individual food recommendations, both Canada’s Food Guide and the DCCP promote healthy dietary patterns, such as the Dietary Approaches to Stop Hypertension (DASH) diet, for its well-established health benefits (24, 25). However, there is limited research examining the alignment of CAN-FOPL with these multiple guidelines and recommendations for healthy eating for Canadians.

Therefore, the objectives of the study were (i) to examine the diet quality of Canadian adults using the CAN-FOPL dietary index system, and (ii) to assess the alignment of CAN-FOPL dietary index system with other dietary index systems (i.e., DCCP, Nutri-score, DASH and HEFI-2019) with HEFI-2019 as the reference standard.

## 2. Methods

### 2.1. Dietary data

Dietary intakes of Canadian adults were assessed using the 2015 Canadian Community Health Survey-Nutrition (CCHS) Public Use Microdata File (26). CCHS is a nationally representative, cross-sectional 24-h dietary recall survey data of Canadians conducted by Statistics Canada (26). CCHS uses a general health questionnaire to collect self-reported sociodemographic, anthropometric, and health status data; and a 24-h dietary recall to collect food and beverage intake of an individual over 24 h with a second recall collected from a subset of participants (26). CCHS includes data from all individuals ≥1 years of age living in private dwellings in the 10 Canadian provinces, excluding full-time members of the Canadian Forces or who lived in the Territories, on reserves and other Indigenous settlements, in some remote areas, or institutions (e.g., prisons or care facilities) (26). Out of 20,487 respondents in the CCHS, data were excluded from the analysis if respondents were under 19 years of age (*n* = 6,568), underweight (body mass index [BMI] <18.5 kg/*m*^2^; *n* = 230), were lactating (*n* = 183), or did not report any food consumption (*n* = 11). The final sample size used for the study was 13,495.

Energy intake (EI) to total energy expenditure (TEE) ratio for each respondent was calculated to identify misreporters, as previously reported (27). TEE was calculated based on age, sex, measured or adjusted self-reported BMI, and physical activity levels using the Institute of Medicine equations (28). Physical activity levels were categorized into sedentary, low active, active, and very active based on the average physical activity per day in minutes, converted from self-reported hours of physical activity per week (26). For respondents that did not disclose any anthropometric information, TEE was assigned based on age, sex, and physical activity levels estimated in the Dietary Guidelines for Americans 2020–2025 (29). EI:TEE ratios of 0.7–1.42 were used to define plausible reporters, while EI:TEE <0.7 and >1.42 were used to define under- and over-reporters, respectively (30).

Foods reported in CCHS were matched to the Canadian Nutrient File database, a generic food composition database of commonly-consumed foods with over 150 nutrients (31).

### 2.2. Canadian front-of-pack labelling (CAN-FOPL) dietary index system

**Figure 1:**
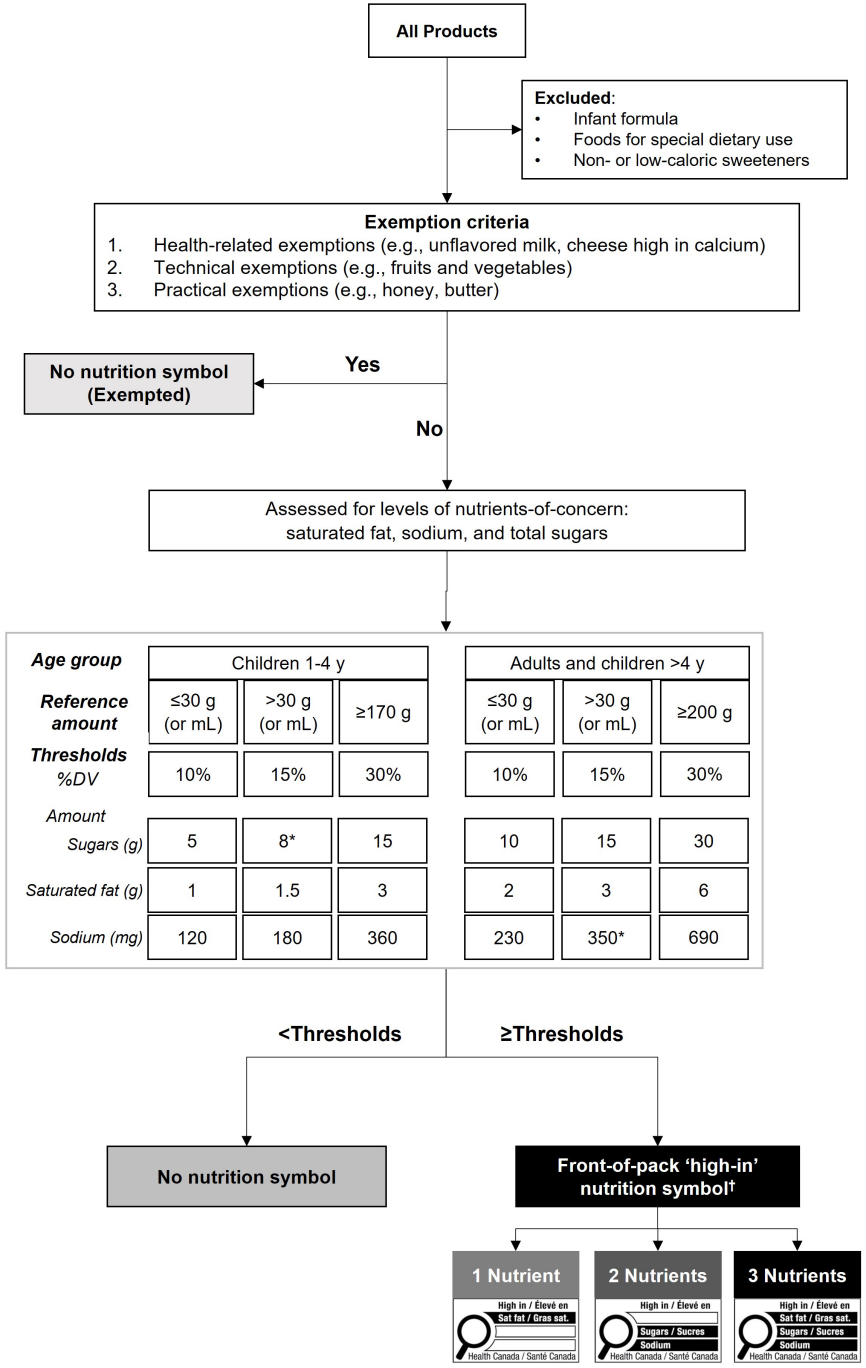
Flow chart of the nutrient profiling model developed according to Canadian front-of-pack labelling regulations. Canadian front-of-pack labelling (CAN-FOPL) regulations (7) were used to categorize all foods in the Canadian Nutrient File, which was matched to foods reported in the 2015 Canadian Community Health Survey. Foods that are not subjected to FOPL regulations (e.g., alcoholic beverages, meal replacements, and non-or low-caloric sweeteners) were excluded. All other foods were assessed against 3 exemption criteria: (i) health-related exemptions for foods that have shown health benefits (e.g., fruits and vegetables, oils high in unsaturated fats); (ii) technical exemptions for foods that are not required to display a Nutrition Facts table (e.g., raw single ingredient meats); and (iii) practical exemptions for foods that are well-known sources of nutrients-of-concern (e.g., honey, butter, table salt). Any foods not exempted from the regulations were assessed for levels of nutrients-of-concern (saturated fat, total sugars, sodium) of percent daily value (%DV) thresholds set based on the target age group and the reference amount (as per Health Canada’s Table of Reference Amounts for Foods (32)) for foods, resulting in six different thresholds. Products meeting or exceeding any of the thresholds of nutrients-of-concern would be required to display a FOP symbol for 1–3 nutrients. ^*^The values are adjusted according to the rounding rules for nutrition labelling information as per *Food and Drugs Regulations* (33). ^†^FOP symbol image was retrieved from *Canada Gazette II* (7). %DV, Percent Daily Value.

Figure 1 shows the flow chart of the nutrient profiling model developed according to Canadian FOPL regulations. FOPL regulations published in *Canada Gazette II* (7) were used to develop a Canadian Front-of-Pack Labelling (CAN-FOPL) nutrient profiling model (8). The model uses exemption criteria (i.e., not assessed for nutrient levels) and thresholds for 3 nutrients-of-public health concern (i.e., saturated fat, sodium, and total sugars) based on age groups (1–4-year-old children; and children over 4 years of age and adults) and reference amounts (foods with a small reference amount ≤30 g or 30 mL; foods with a reference amount >30 g or 30 mL; foods with a reference amount ≥170 g for 1-4-year-old children; and foods with a reference amount ≥200 g for children over 4 years of age and adults) to classify foods into 5 categories (Exempted from FOPL regulations; Not display a symbol due to <thresholds; Display a symbol for 1 nutrient; Display a symbol for 2 nutrients; Display a symbol for 3 nutrients). Based on FOPL regulations, 3 types of foods and beverages are exempted from displaying a “high-in” symbol, regardless of their nutrient levels: (i) foods that have shown to have recognized health protection benefits (e.g., unflavored milk, eggs, fruits and vegetables, cheese high in calcium); (ii) foods that are exempted from carrying a Nutrition Facts table (e.g., single ingredient meats, foods sold in very small packages); and (iii) foods that are known sources of the target nutrients (e.g., table sugar, honey, salt, butter) (7).

The CAN-FOPL nutrient profiling model was used to construct the CAN-FOPL dietary index system using a 2-step approach, as previously reported (18). First, foods and beverages categorized according to the CAN-FOPL nutrient profiling model were assigned a point on a scale of 100 (“more healthy”) to 0 (“less healthy”) in 25-point increments in a descending order. In other words, foods and beverages in the first category (i.e., exempted from CAN-FOPL regulations) were assigned 100 points, 75 points for the second category (i.e., below the threshold levels for all 3 nutrients), 50 points for the third category (i.e., meet and/or exceed threshold levels for 1 nutrient), 25 points for the fourth category (i.e., meet and/or exceed threshold levels for 2 nutrients), and 0 point for the fifth category (i.e., meet or exceed threshold levels for 3 nutrients). Second, the points from the CAN-FOPL nutrient profiling model were pooled for each participant, weighted by the proportion of energy contributed by each food to get an individual dietary index score (*Equation 1*), as previously reported (18).

(1) $$\begin{array}{c} \mathrm{CAN}-\mathrm{FOPL dietary}\\ \mathrm{index score} \end{array}=\frac{\sum_{i=1}^{n}\left(\mathrm{CAN}-{\mathrm{FOPL point}}_{i}\right)\times E_{i}}{\sum_{i=1}^{n}E_{i}}$$

“*where standardized CAN-FOPL point_i_ is the assigned point based on the CAN-FOPL nutrient profiling model categories for each individual food or beverage consumed, and E_i_ is the energy intake from that food or beverage*”.

### 2.3. Application of dietary index systems

Dietary data from CCHS were assigned scores based on the five dietary index systems: CAN-FOPL, DCCP, Nutri-score, DASH and HEFI-2019. With CAN-FOPL regulations expected to influence the diets of Canadians, their alignment with other guidelines for healthy eating for Canadians (i.e., DCCP, DASH, and Canada’s Food Guide) and other FOPL system shown to have positive association with NCDs (i.e., Nutri-score) were examined. The HEFI-2019 (dietary index system based on Canada’s Food Guide) was used as the reference standard in the analysis, as Canada’s Food Guide is the current national dietary guidelines for all Canadians over 2 years of age to improve health, meet nutrient needs, and reduce risk of NCDs (10).

The scoring methods for the DCCP, the Nutri-score, DASH, and HEFI-2019 dietary index systems are described in detail in Supplementary methods. Briefly, the DCCP nutrient profiling model assesses foods and beverages for their alignment with the DCCP by assigning points for their macronutrient or meal quality, saturated and trans fat content, and added sugar content, while beverages are assigned points based on the beverage type, saturated fat, and added sugar content (18) to classify foods and beverages into one of 3 categories: “most aligned,” “partially aligned,” and “least aligned.” The Nutri-score assigns points to foods and beverages for their energy and nutrient content (total sugars, saturated fats, and sodium as “negative” nutrients and fiber and protein as “positive” nutrients), energy and nutrient content (total sugars, saturated fats, and sodium as “negative” nutrients and fiber and protein as “positive” nutrients), and the amount of fruits, vegetables, nuts, or legumes; then sums up the points to classify foods and beverages into one of 5 categories: Grade A (“more healthy”), Grade B, Grade C, Grade D, and Grade E (“less healthy”). Similar to the algorithm for the CAN-FOPL dietary index scores, the DCCP, and the Nutri-score dietary index scores were calculated by assigning points to foods and beverages from nutrient profiling models underlying each system, adjusting the points by the proportion of energy contribution from each food or beverage, and summing up the energy-adjusted points for a final dietary index score.

The DASH (34) and the HEFI-2019 (13) assign dietary index scores using foods and beverages consumed in a 24-h period to reflect adherence to the DASH diet and Canada’s Food Guide, respectively. The DASH dietary index system (34) assigns points for 9 dietary components identified in the DASH diet: Fruit, Vegetables, Whole Grains, Dairy Products, Plant Proteins, Animal Proteins, Added Sugars, Sodium, and Saturated Fat. The HEFI-2019 scores diets against 10 key recommendations of Canada’s Food Guide: Vegetables and fruits, Whole-grain foods, Grain foods ratio, Protein foods, Plant-based protein foods, Beverages, Fatty acids ratio, Saturated fats, Free sugars, and Sodium. All dietary index scores were converted into a 100-score system to standardize the scores, with 0 indicating “Least aligned” or “Least healthy” to 100 indicating “Most aligned” or “Most healthy.”

### 2.4. Statistical analysis

All statistical analyses were performed using SAS (version 9.4, SAS Institute Inc., Cary, NC, United States). Mean nutrient intakes were examined across quintiles using PROC SURVEYREGS, adjusted for sex, age, energy intake, and misreporter status. Misreporter status was selected as a confounding variable, as it has been shown to adjust for implausible recalls and selective misreporting of healthy vs. unhealthy foods; and indirectly adjust for socioeconomic characteristics correlated with misreporting status, including education and smoking status (35). Linear trends of energy and nutrient intakes across quintile groups using the CAN-FOPL, the DCCP, and the Nutri-score dietary index scores were assessed by assigning participants the median value in each quintile and modeling it as a continuous variable. Diet quality of Canadians using the DASH (36) and HEFI-2019 (12, 13) have been reported elsewhere. All estimates were bootstrapped using the balanced repeated replication technique with 500 replicates of population survey weights provided by Statistics Canada (37) to ensure representative population-level estimates. Based on a previous analysis examining diet quality using CCHS 2.2 with a large sample size and bootstrapping technique (35, 36). *p*-trend <0.0001 was considered significant.

Weighted Pearson’s correlation coefficients were determined between all dietary index scores to assess the alignment between dietary index scores. To evaluate agreement between pairs of dietary index scores, total scores from each index were divided into quintiles, and the agreement of the sample falling into quintile categories for pairs of indices was examined using weighted *κ* statistic (95% CI), as follows: 0.01–0.20 “slight”; 0.21–0.40 “fair”; 0.41–0.60 “moderate”; 0.61–0.80 “substantial”; and 0.81–0.99 “near perfect” (38). *p*-value < 0.05 was considered significant. Bland-Altman plots between all dietary index systems were used to visually inspect the level of agreement.

## 3. Results

### 3.1. Dietary index scores and nutrient intakes

About 50% of the respondents were females with a mean age [95% CI] of 49.3 years [48.8, 49.8]. Based on the reported energy intake to total energy expenditure ratio, 50% of the respondents were identified as plausible, 44% as under-reporters and 6% as over-reporters. Detailed participant characteristics can be found elsewhere (8).

The mean standardized dietary index scores [95% CI] for CAN-FOPL, DCCP, Nutri-score, DASH, and HEFI-2019 were 73.0 [72.8, 73.2], 64.2 [64.0, 64.3], 54.9 [54.7, 55.1], 51.7 [51.4, 51.9], and 54.3 [54.1, 54.6], respectively. Table 1 and Supplementary Tables S1, S2 show nutrient intakes by quintiles of the CAN-FOPL, the DCCP, and the Nutri-score dietary index scores, respectively. Moving from quintile 1 (“Least healthy”) to quintile 5 (“Most healthy”) of the CAN-FOPL dietary index scores, intakes of protein, fiber, vitamin A, vitamin C, vitamin D, and potassium increased, while intakes of energy, saturated fat, and sodium decreased (all *p* < 0.0001). There was no linear relation for total fat and calcium intakes (*p* = 0.003 and *p* = 0.024, respectively) across quintile groups using the CAN-FOPL dietary index scores. Moving from quintile 1 to quintile 5 of the DCCP dietary index scores, intakes of protein, fiber, vitamin A, vitamin C, vitamin D, iron, and potassium increased, while intakes of total energy, total and saturated fat, free sugars, and sodium decreased (all *p* < 0.0001). There was no linear relation for calcium intakes (*p* = 0.0005) across quintile groups using the DCCP dietary index scores. Moving from quintile 1 to quintile 5 of the Nutri-score dietary index scores, intakes of carbohydrates, fiber, protein, vitamin A, iron, and potassium increased, while intakes of energy, total and saturated fat, total and free sugars, and calcium decreased (all *p* < 0.0001). There was no linear relation for vitamin D intakes (*p* = 0.10) across quintile groups using the Nutri-score dietary index scores.

**Table 1:** Energy and nutrient intakes across quintile groups using the Canadian Front-of-pack labelling (CAN-FOPL) dietary index system.

	Quintile 1 (“least healthy”)	Quintile 2	Quintile 3	Quintile 4	Quintile 5 (“most healthy”)	*p*-trend
*n*	2,699	2,699	2,699	2,699	2,699	
CAN-FOPL dietary index score*	54.5 [53.5, 55.6]	68.0 [67.7, 68.3]	74.6 [74.5, 74.7]	80.0 [79.9, 80.1]	87.5 [87.2, 87.8]	
Energy (kcal)	2,503 [2,431, 2,575]	2,412 [2,361, 2,464]	2,387 [2,345, 2,429]	2,350 [2,299, 2,401]	2,292 [2,244, 2,340]	<0.0001
Total fat (% to total energy)	34.7 [34, 35.4]	33.6 [32.5, 34.8]	33.4 [31.8, 35]	32.9 [32.1, 33.6]	33.6 [32.1, 35.2]	0.003
Saturated fat (% to total energy)	12.1 [11.7, 12.6]	11.4 [11, 11.8]	10.7 [10.2, 11.1]	10.4 [10, 10.9]	10.0 [9.5, 10.5]	<0.0001
Protein (% to total energy)	15.1 [14.3, 15.9]	15.7 [15.2, 16.1]	16.2 [15.8, 16.7]	17.9 [17.4, 18.4]	19.4 [18.5, 20.2]	<0.0001
Carbohydrates (% to total energy)	47.1 [46, 48.3]	48.6 [47.2, 50]	48.6 [46.6, 50.7]	47.3 [45.8, 48.9]	44.8 [43, 46.7]	<0.0001
Fiber (g/1,000 kcal)	7.4 [7.1, 7.8]	8.7 [8.3, 9.2]	9.7 [8.9, 10.6]	10.2 [9.5, 10.9]	10.5 [10.1, 11]	<0.0001
Total sugars (% to total energy)	18.3 [15.9, 20.8]	19.6 [18.2, 21]	18.2 [16.6, 19.9]	17.4 [15.7, 19]	17.8 [15.9, 19.6]	<0.0001
Free sugars (% to total energy)	10.9 [8.5, 13.3]	11.6 [10.3, 12.8]	9.6 [8.3, 10.9]	8.0 [6.4, 9.6]	6.8 [5.1, 8.5]	<0.0001
Calcium density (mg/1,000 kcal)	408.4 [391.2, 425.5]	417.8 [402.3, 433.3]	430.9 [411.6, 450.1]	425.9 [408.3, 443.5]	403.1 [385.8, 420.4]	0.024
Vitamin A density in RAE (μg/1,000 kcal)	261.7 [239.6, 283.8]	316.7 [290.9, 342.4]	359.8 [290.6, 429]	381.5 [346.1, 417]	457.5 [413.8, 501.1]	<0.0001
Vitamin C density (mg/1,000 kcal)	37.6 [31.5, 43.7]	53.1 [47.1, 59.1]	54.6 [50.5, 58.7]	57.6 [52.3, 62.9]	58.7 [48.1, 69.3]	<0.0001
Vitamin D density (μg/1,000 kcal)	1.9 [1.7, 2]	2.3 [2.1, 3.1]	2.6 [2.4, 2.8]	2.9 [2.6, 3.1]	3.2 [2.9, 3.6]	<0.0001
Sodium density (mg/1,000 kcal)	1,619.3 [1,566.7, 1,671.9]	1,515.5 [1,434.5, 1,596.6]	1,465.5 [1,416.8, 1,514.2]	1,370.3 [1,309.2, 1,431.3]	1225.6 [1,181.5, 1,269.7]	<0.0001
Iron density (μg/1,000 kcal)	6.3 [6.1, 6.5]	6.4 [6.1, 6.7]	6.9 [6.6, 7.1]	6.9 [6.6, 7.2]	6.7 [6.5, 6.9]	<0.0001
Potassium density (mg/1,000 kcal)	1,235.0 [1,188.5, 1,281.5]	1,334.1 [1,302.9, 1,365.4]	1,427.6 [1,393.3, 1,461.9]	1,521.1 [1,458.2, 1,583.9]	1,723.3 [1,668.5, 1,778.2]	<0.0001

*n* = 13,495; values represent means [95% CI]. Estimates are weighted least squares means from a regression model adjusted for age, sex, misreporting status (under-reporters, plausible reporters and over-reporters), and energy with bootstrapping. *p*-trends were estimated in their continuous form and represent the *p*-value associated with the linear regression coefficient. *p*-trend < 0.0001 was considered significant. *CAN-FOPL dietary index scores were calculated by assigning points to foods and beverages categorized using the CAN-FOPL nutrient profiling model, adjusting the points by the proportion of energy contribution from each food and beverage, and then summing up the energy-adjusted points for a final score. The dietary index score was standardized on a scale of 0 (“least healthy”) to 100 (“most healthy”). CAN-FOPL, Canadian Front-of-pack labelling; RAE, Retinol Activity Equivalents.

### 3.2. Relationship between dietary index systems

Table 2 shows weighted Pearson’s correlations between dietary index scores. The CAN-FOPL dietary index scores showed poor to moderate correlation with the DCCP, the Nutri-score, the DASH, and the HEFI-2019 (*r* = 0.242–0.545). The DCCP, the Nutri-Score, and the DASH dietary index scores were moderately correlated with the HEFI-2019 (*r* = 0.615–0.640, *p* < 0.0001).

**Table 2:** Weighted Pearson’s correlations between dietary index systems.

	CAN-FOPL	DCCP	Nutri-score	DASH	HEFI-2019
CAN-FOPL	1.000	0.545	0.444	0.242	0.401
DCCP		1.000	0.702	0.420	0.640
Nutri-score			1.000	0.343	0.619
DASH				1.000	0.615
HEFI-2019					1.000

*n* = 13,495. Values are correlation coefficient (r); all *p*-values were significant (*p* < 0.0001). CAN-FOPL, Canadian Front-of-Pack Labelling; DASH, Dietary Approaches to Stop Hypertension Diet; DCCP, Diabetes Canada Clinical Practice Guidelines; HEFI-2019, Healthy Eating Food Index 2019.

Table 3 and Supplementary Tables S4, S5 show the agreement between quintile combinations of CAN-FOPL and other dietary index systems, including the reference standard (i.e., HEFI-2019). The CAN-FOPL dietary index scores showed fair agreement with the DCCP and the Nutri-score (*k* = 0.30–0.38) with over 65% of the total sample identified as discordant pairs (i.e., “Less healthy” in one system and “More healthy” in another system). The CAN-FOPL, the DCCP and the Nutri-score showed slight agreement with the DASH (*к* = 0.05–0.07) with over 75% of the total sample as discordant pairs. The CAN-FOPL showed fair agreement with the HEFI-2019 (*к* = 0.26 [0.25, 0.27]); however, both the DCCP and the Nutri-score showed moderate agreement with the HEFI-2019 (*к* = 0.44 [0.43, 0.46] and *к* = 0.42 [0.42, 0.44], respectively) with about 60% of the total sample as discordant pairs.

**Table 3:** Agreement between quintile combinations of computed Canadian Front-of-pack labelling and other dietary index systems.

		CAN-FOPL					Discordant pairs*, *n* (%)	Weighted к^†^ [95% CI]
		Q1	Q2	Q3	Q4	Q5		
DCCP	Q1	9.6	5.6	2.9	1.3	0.5	8,852 (65.6%)	0.38 [0.36, 0.39]
	Q2	5.0	5.6	4.6	3.2	1.6		
	Q3	3.2	4.0	4.9	4.7	3.3		
	Q4	1.8	3.1	4.3	5.2	5.5		
	Q5	0.5	1.7	3.2	5.5	9.1		
Nutri-score	Q1	8.7	5.2	2.9	1.8	1.5	9,217 (68.3%)	0.30 [0.29, 0.31]
	Q2	5.0	5.1	4.4	3.3	2.2		
	Q3	3.2	4.3	4.7	4.5	3.3		
	Q4	2.2	3.4	4.4	5.1	4.9		
	Q5	0.9	2.1	3.6	5.3	8.1		
DASH	Q1	18.8	16.9	15.5	15	15.1	10,431 (77.3%)	0.05 [0.05, 0.06]
	Q2	1	2.1	2.9	2.9	2.7		
	Q3	0.2	0.7	0.9	1.2	1.2		
	Q4	0.1	0.2	0.5	0.6	0.7		
	Q5	<0.1	0.1	0.2	0.3	0.3		
HEFI-2019^‡^	Q1	7.8	4.9	3.4	2.3	1.6	9,500 (70.4%)	0.26 [0.25, 0.27]
	Q2	5.0	4.7	4.1	3.5	2.6		
	Q3	3.6	4.6	4.5	4.0	3.3		
	Q4	2.6	3.6	4.2	4.8	4.8		
	Q5	1.1	2.1	3.7	5.3	7.8		

*n* = 13,495. Increasing quintiles (Q) indicate higher scores (i.e., “healthier” diet quality). Each cell includes the proportion (%) of the total sample falling into the respective quintile combinations. Shaded cells indicate concordant pairs (i.e., samples falling into the same quintile according to the two examined dietary index systems) with 20% in each cell representing perfect agreement, while non-shaded cells indicate discordant pairs (i.e., samples identified as “Less healthy” in one dietary system and “More healthy” in another dietary index system). *Discordant pairs are presented as the total number of identified samples and the proportion (%) of the total sample. ^†^Agreement between dietary index scores were assessed using weighted *κ* statistic, where: 0.01–0.20 represented “slight” agreement; 0.21–0.40 “fair”; 0.41–0.60 “moderate”; 0.61–0.80 “substantial”; and 0.81–0.99 “near perfect” (38). ^‡^HEFI-2019 was set as the reference standard. CAN-FOPL, Canadian Front-of-Pack Labelling; DASH, Dietary Approaches to Stop Hypertension Diet; DCCP, Diabetes Canada Clinical Practice Guideline; HEFI, Healthy Eating Food Index.

Supplementary Figures S1–S3 show Bland-Altman plots comparing dietary index scores from different systems. CAN-FOPL dietary index scores showed relatively good agreement with DCCP (mean difference [95% limits of agreement] = 8.8 [−11.3, 29.0]), but poor agreement with Nutri-score with wide variability. CAN-FOPL showed a wide mean difference compared to the DASH (21.3 [−9.0, 51.7]), while the DCCP and the Nutri-score showed smaller mean difference compared to the DASH but wide variability (12.5 [–12.4, 37.4] and 3.2 [−27.0, 33.4], respectively). Similarly, when compared to the reference standard (i.e., HEFI-2019), the CAN-FOPL showed a wide mean difference and variability (18.7 [−10.1, 47.5]), while the DCCP and the Nutri-score showed smaller mean differences with wide variability (9.8 [−12.4, 32.0] and 0.5 [−23.8, 24.8], respectively).

## 4. Discussion

The objectives of the study were to describe the diet quality of Canadians using the CAN-FOPL dietary index system, and then compare the results to other dietary index systems (i.e., DCCP, Nutri-score, DASH, and HEFI-2019), using the HEFI-2019 as the reference standard. Although the CAN-FOPL dietary index system discriminated diet quality using nutrients-of-public health concern, it did not align well with other dietary systems underpinning health policies and recommendations. On the contrary, the DCCP and the Nutri-score dietary index systems showed better ability to discriminate diet quality using more nutrients-of-public health concern and nutrients-to-encourage, and moderate alignment with the HEFI-2019 (a dietary index scoring system based on Canada’s Food Guide, which also includes foods to encourage).

Among the five examined dietary index systems, the CAN-FOPL showed the highest mean score for Canadians of 73 (vs. 52–64 for others), with a wide mean difference and the limits of agreement compared with other dietary index systems prominently shown in Bland-Altman plots. The findings are likely related to the very low consumption of foods that are rated as “least healthy” (i.e., display a front-of-pack symbol for meeting and/or exceeding all 3 nutrients-of-concern), and the high rating of foods in the exemption criteria (i.e., 100 out of 100) according to CAN-FOPL regulations. In fact, Canadian adults consumed <1% of total energy from foods that would display a front-of-pack symbol for all 3 nutrients-of-concern, while 35% of energy intakes came from foods that would be exempted from CAN-FOPL regulations (8), thus limiting its ability to discriminate among a wide range of foods of varying nutritional quality. The nature of the CAN-FOPL nutrient profiling model, which only focuses on nutrients-of-concern, may contribute to lower discriminatory ability to further differentiate the healthfulness of foods and diet quality, compared to the other examined nutrient profiling models (i.e., DCCP and Nutri-score), which take both nutrient- and food-based approach to rank the healthfulness of foods. In other words, when CAN-FOPL regulations are implemented, Canadians would only be exposed to two conditions (with or without a front-of-pack symbol) and will need to determine the healthfulness of foods among these two conditions. Whether food meets other healthy dietary guidelines, such as Canada’s Food Guide or the DASH diet, will not be readily available to consumers. To address this gap, in addition to a mandatory FOPL system for nutrients-of-concern (saturated fat, sugars, and sodium), Israel introduced a voluntary FOPL system to indicate foods that align with the Mediterranean Diet (5). Other voluntary FOPL systems have been shown to improve the purchasing intentions and behaviours of consumers (39), which may be helpful for Canadians to select ‘healthier’ foods among those foods that do not have a front-of-pack symbol.

Although CAN-FOPL showed some discriminatory ability to assess diet quality, it was poorly aligned with the reference standard, HEFI-2019. Consistent with our findings, CAN-FOPL dietary index system based on the proposed regulations showed a similar ability to discriminate nutrients-to-limit, including saturated fat, added sugars, and sodium; and showed a negative association with fasting blood glucose (i.e., lower blood glucose associated with “more healthy” score) using a French cohort data (18). Interestingly, we saw no difference in calcium intakes by CAN-FOPL quintiles with improved diet quality despite dairy products high in calcium, one of the main sources of calcium and vitamin D for Canadians (40, 41), being exempted from CAN-FOPL regulations. The findings, however, may be related to a high prevalence of inadequate calcium intakes among Canadians. Based on the same CCHS 2015 dietary data, almost 70% of calcium supplement non-users have been found to have calcium intakes below the estimated average requirements (similar calcium intakes from food sources among supplement users), with over 90% of older adults (≥71 y) having inadequate intake levels (40). To help Canadians meet their nutrient needs and encourage the consumption of healthy foods, additional public health measures, including voluntary fortification may need to be explored. The poor alignment between CAN-FOPL and HEFI-2019 also signals a potential need for further public health interventions to help Canadians follow healthy eating guidelines. The most up-to-date Canada’s Food Guide in 2019 included recommendations on food choices and healthy eating habits, and employed various communication strategies to reach the public, such as user-friendly implementation tools (e.g., recipes) and consistent reminders on healthy eating (e.g., subscription system to receive monthly updates) (9, 10); yet, its effectiveness on the population health has not been well documented. To assess the effectiveness of these public health guidelines and regulations, using CCHS-2015 as a baseline, future studies examining the diet quality of Canadians post-regulations are needed.

Interestingly, the DCCP dietary index system showed a greater discriminatory ability to assess diet quality compared to the CAN-FOPL dietary index system with a moderate association with the HEFI-2019. Despite the lack of sodium assessment built into the DCCP nutrient profiling model, it discriminated sodium intakes when transformed as a dietary index system, as similarly shown with a previous study employing the DCCP model to examine the diet quality of a French cohort (18). It is possible that the food-based recommendations in the DCCP model are related to lower sodium content, resulting in foods with low sodium content scoring higher in the nutrient profiling model. For instance, the DCCP model includes a step to allocate a score for the processing level of foods, where processed and ultra-processed foods, defined using the NOVA classification (42, 43), would get a lower score compared with minimally and unprocessed foods. Since sodium content is typically high in processed foods, as sodium ingredients are used as a preservative and/or a flavor enhancer (44), the processing level assessment step may act as a proxy for sodium content in foods to help discriminate sodium intake at the dietary level. Further, DCCP was moderately aligned with other dietary index systems, including CAN-FOPL and HEFI-2019. The alignment between DCCP and HEFI-2019 are likely related to the fact that Canada’s Food Guide, the guidelines on which HEFI-2019 is based, reflects the most up-to-date summary of evidence that supports healthy eating patterns that could lower the risk of NCDs, including diabetes (10). Emerging evidence suggests multiple healthy eating patterns with different components can lead to health benefits (45); therefore, promotion of multiple healthy eating guidelines respecting individual variation would be beneficial for realistic and long-term adherence.

Despite the wide recommendation of the DASH diet in many clinical guidelines, including the DCCP and Canada’s Food Guide, all dietary index systems showed poor alignment with the DASH diet. The findings are, in part, likely related to low overall adherence to the DASH diet among Canadians, corroborated by the greatest agreement seen between the least aligned quintile of the DASH dietary index system with all quintiles of the examined dietary index systems. In fact, a previous study showed only about 50% of adherence to the DASH diet among Canadians based on the population dietary survey from 2004 and 2015 (36). The differing emphasis on food components may also contribute to the differences in these diet assessment tools. The DASH dietary index system consists of 9 equally weighted components with some that are not frequently consumed in the Canadian diet (e.g., whole grains and plant proteins) (36), contributing to the low adherence scores and the poor association with other dietary index systems. Although many of the components of the DASH diet are included in the examined dietary index systems (i.e., DCCP and HEFI-2019), their contribution to the overall dietary index scores differs. For example, consumption of plant-based protein contributes to 1/9^th^ of the total score for the DASH dietary index scores (34) while the DCCP dietary index model takes intakes relative to total energy into account (18) and the HEFI-2019 allocates about 6% (5/80) of the total point to plant-based food consumption (13). To help support intakes of foods- (e.g., whole grains) and nutrients-to-encourage (e.g., fiber), additional support is needed at both population and individual levels.

Although this is the first study to date, examining the alignment of the recently published CAN-FOPL with other FOPL systems and dietary guidelines, a few limitations must be noted. First, we could not assess its association with mortality or disease risk due to the nature of the CCHS data. Previous studies have shown that dietary index systems can quantify diet quality and its association with markers of cardiovascular disease risk using prospective cohorts (18, 20, 23, 46). However, CCHS, one of the only publicly available Canadian dietary data, is a cross-sectional survey without any clinical biomarkers of disease risk. As Canada implements its FOPL regulations, a robust monitoring and evaluation plan to measure their short- and long-term impact on dietary patterns and risk of mortality and disease is needed. Second, the present analysis was conducted using single-day dietary data. Although single-day, 24-h recall can be reflective of the usual intakes at a population level, suitable for the current analysis at a population level, it can be affected by within-person variation due to day-to-day variation of food intakes (47), and cannot be used for assessment at the individual level. Third, these findings do not necessarily indicate the strength of one dietary index system over another, *per se*, but rather incorporate the inherent challenges and complexity in the assessment of diet quality. The DASH and the HEFI-2019 dietary index systems evaluate adherence to different diets using an individual’s overall dietary intake in a specific time period, while other dietary index systems developed from nutrient profiling models (i.e., CAN-FOPL, DCCP, and Nutri-score) quantify the diet quality based on the quality of individual foods consumed and their proportions. The assumptions made in the development of the dietary index systems may have affected the observed associations (48). However, at the population level, these dietary models provide insight into potential gaps in nutritional policy and/or guidelines and how diet quality indices compare with one another.

## 5. Conclusion

Our findings show that CAN-FOPL regulations, which only focuses on nutrients-to-limit, rated the dietary quality of Canadian adults to be healthier than other dietary index systems, and it may be used to examine the quality of dietary intakes of Canadians. Despite the good agreement between the CAN-FOPL and the DCCP, wide differences with the DASH and the HEFI-2019 suggest a potential gap in Canadian FOPL regulations, particularly in supporting consumption of “more healthy” foods. Although FOPL regulations will go a long way towards helping Canadians avoid less healthy foods, further public health guidelines and recommendations are warranted to promote the consumption of “healthy” foods and/or adherence to a healthy diet; and robust evaluation and monitoring plan are needed to assess the effectiveness of FOPL regulations in achieving their objectives.

## Data availability statement

The data underlying the results presented in the study are available from Statistics Canada: https://www150.statcan.gc.ca/n1/en/catalogue/82M0024X2018001.

## Ethics statement

Ethical review and approval was not required for the study of human participants in accordance with the local legislation and institutional requirements. As a secondary data analysis, written informed consent from the participants was not required in this study in accordance with the national legislation and the institutional requirements.

## Author contributions

JJL, MA, CJ, WYL, and MRL contributed to conception and design of the study and interpreted the findings. JJL, AN, and MA organized the database. JJL and MA performed the statistical analysis. All authors contributed to the article and approved the submitted version.

## Funding

This research was funded by the Sanofi-Pasteur-University of Toronto-Universite Paris-Descartes International Collaborative Research Pilot and Feasibility Program (Grant #507462).

## Conflict of interest

The authors declare that the research was conducted in the absence of any commercial or financial relationships that could be construed as a potential conflict of interest.

## Publisher’s note

All claims expressed in this article are solely those of the authors and do not necessarily represent those of their affiliated organizations, or those of the publisher, the editors and the reviewers. Any product that may be evaluated in this article, or claim that may be made by its manufacturer, is not guaranteed or endorsed by the publisher.
