# Corrigendum: Examining the diet quality of Canadian adults and the alignment of Canadian front-of-pack labelling regulations with other front-of-pack labelling systems and dietary guidelines

**DOI:** 10.3389/fpubh.2024.1448853

**Published:** 2024-08-06

**Authors:** Jennifer J. Lee, Mavra Ahmed, Chantal Julia, Alena Praneet Ng, Laura Paper, Wendy Y. Lou, Mary R. L'Abbé

**Affiliations:** ^1^Department of Nutritional Sciences, Temerty Faculty of Medicine, University of Toronto, Toronto, ON, Canada; ^2^Joannah and Brian Lawson Centre for Child Nutrition, University of Toronto, Toronto, ON, Canada; ^3^Sorbonne Paris Nord University, INSERM, INRAE, CNAM, Nutritional Epidemiology Research Team (EREN), Epidemiology and Statistics Research Center, University of Paris (CRESS), Bobigny, France; ^4^Public Health Department, Avicenne Hospital, AP-HP, Bobigny, France; ^5^Nutritional Epidemiology Surveillance Team (ESEN), Santé Publique France, The French Public Health Agency, Bobigny, France; ^6^Biostatistics Division, Dalla Lana School of Public Health, University of Toronto, Toronto, ON, Canada

**Keywords:** front-of-pack, FOPL, dietary patterns, nutrient profiling, HEFI, Nutri-score, DASH, DCCP

In the published article, there was an error in Table 3 as published. Incorrect values were entered into the “discordant pairs” and “weighted k” columns. The corrected Table 3 and its caption “Agreement between quintile combinations of computed Canadian Front-of-pack labelling and other dietary index systems” appear below.

**Table 3:** Agreement between quintile combinations of computed Canadian Front-of-pack labelling and other dietary index systems.

		**CAN-FOPL**					Discordant pairs^*^, *n* (%)	**Weighted k^†^ [95% CI]**
		**Q1**	**Q2**	**Q3**	**Q4**	**Q5**		
DCCP	Q1	9.6	5.6	2.9	1.3	0.5	8,852 (65.6%)	0.38 [0.36, 0.39]
	Q2	5.0	5.6	4.6	3.2	1.6		
	Q3	3.2	4.0	4.9	4.7	3.3		
	Q4	1.8	3.1	4.3	5.2	5.5		
	Q5	0.5	1.7	3.2	5.5	9.1		
Nutri-score	Q1	8.7	5.2	2.9	1.8	1.5	9,217 (68.3%)	0.30 [0.29, 0.31]
	Q2	5.0	5.1	4.4	3.3	2.2		
	Q3	3.2	4.3	4.7	4.5	3.3		
	Q4	2.2	3.4	4.4	5.1	4.9		
	Q5	0.9	2.1	3.6	5.3	8.1		
DASH	Q1	18.8	16.9	15.5	15.0	15.1	10,431 (77.3%)	0.05 [0.05, 0.06]
	Q2	1.0	2.1	2.9	2.9	2.7		
	Q3	0.2	0.7	0.9	1.2	1.2		
	Q4	0.1	0.2	0.5	0.6	0.7		
	Q5	< 0.1	0.1	0.2	0.3	0.3		
HEFI-2019^‡^	Q1	7.8	4.9	3.4	2.3	1.6	9,500 (70.4%)	0.26 [0.25, 0.27]
	Q2	5.0	4.7	4.1	3.5	2.6		
	Q3	3.6	4.6	4.5	4.0	3.3		
	Q4	2.6	3.6	4.2	4.8	4.8		
	Q5	1.1	2.1	3.7	5.3	7.8		

*n* = 13,495. Increasing quintiles (Q) indicate higher scores (i.e., “healthier” diet quality). Each cell includes the proportion (%) of the total sample falling into the respective quintile combinations. Shaded cells indicate concordant pairs (i.e., samples falling into the same quintile according to the two examined dietary index systems) with 20% in each cell representing perfect agreement, while non-shaded cells indicate discordant pairs (i.e., samples identified as “Less healthy” in one dietary system and “More healthy” in another dietary index system). ^*^Discordant pairs are presented as the total number of identified samples and the proportion (%) of the total sample. ^†^Agreement between dietary index scores were assessed using weighted κ statistic, where: 0.01–0.20 represented “slight” agreement; 0.21–0.40 “fair”; 0.41–0.60 “moderate”; 0.61–0.80 “substantial”; and 0.81–0.99 “near perfect” (38). ^‡^HEFI-2019 was set as the reference standard. CAN-FOPL, Canadian Front-of-Pack Labelling; DASH, Dietary Approaches to Stop Hypertension Diet; DCCP, Diabetes Canada Clinical Practice Guideline; HEFI, Healthy Eating Food Index.

In the published article, there was an error during the revision process. The weighted k statistic results were incorrectly represented. A correction has been made to Results, *Relationship between Dietary Index Systems*, Paragraph 2. This sentence previously stated:

“The CAN-FOPL dietary index scores showed slight agreement with the DCCP and the Nutri-score (k = 0.30–0.38) with over 65% of the total sample identified as discordant pairs (i.e., “Less healthy” in one system and “More healthy” in another system).”

The corrected sentence appears below:

“The CAN-FOPL dietary index scores showed fair agreement with the DCCP and the Nutri-score (k = 0.30–0.38) with over 65% of the total sample identified as discordant pairs (i.e., “Less healthy” in one system and “More healthy” in another system).”

The authors apologize for the error and state that this does not change the scientific conclusions of the article in any way. The original article has been updated.

