# Supplementary material for: Examining the diet quality of Canadian adults and the alignment of Canadian front-of-pack labelling regulations with other front-of-pack labelling systems and dietary guidelines
Source: Front Public Health. 2023 Jun 23;11:1168745. doi: 10.3389/fpubh.2023.1168745 (PMC10326271; doi:10.3389/fpubh.2023.1168745)
Supplement: Supplementary file 1 [file Data_Sheet_1.pdf]

Supplementary Material

Examining the diet quality of Canadian adults and the alignment of Canadian front-of-pack labelling regulations with other front-of-pack labelling systems and dietary guidelines

Jennifer J. Lee[1], Mavra Ahmed[1,2], Chantal Julia[3,4], Alena (Praneet) Ng[1], Laura Paper[3,5], Wendy Y. Lou[7], Mary R. L'Abbé[1*]

[1]Department of Nutritional Sciences, Temerty Faculty of Medicine, University of Toronto, Toronto, ON, Canada

[2]Joannah and Brian Lawson Centre for Child Nutrition, University of Toronto, Toronto, ON, Canada

[3]Sorbonne Paris Nord University, INSERM, INRAE, CNAM, Nutritional Epidemiology Research Team (EREN), Epidemiology and Statistics Research Center, University of Paris (CRESS), Bobigny, France

[4]Public Health Department, Avicenne Hospital, AP-HP, Bobigny, France

[5]Nutritional Epidemiology Surveillance Team (ESEN), Santé Publique France, The French Public Health Agency, Bobigny, France

[6]Biostatistics Division, Dalla Lana School of Public Health, University of Toronto, Toronto, ON, Canada

*Correspondence: Mary R. L'Abbé, mary.labbe@utoronto.ca

Table of Contents

1 Supplemental Methods 3

1.1 Diabetes Canada Clinical Practice Guidelines (DCCP) Dietary Index System 3

1.2 Nutri-score Dietary Index System 3

1.3 Dietary Approaches to Stop Hypertension (DASH) Dietary Index System 4

1.4 Healthy Eating Food Index-2019 (HEFI-2019) Dietary Index System 4

2 Supplementary Table 1. Energy and nutrient intakes across quintile groups using the Diabetes Canada Clinical Practice Guidelines (DCCP) dietary index system 5

3 Supplementary Table 2. Energy and nutrient intakes across quintile groups using the Nutri-score dietary index system 6

4 Supplementary Table 3. Agreement between quintile combinations of the Dietary Approaches to Stop Hypertension (DASH).and other dietary index systems. 7

6 Supplementary Table 4. Agreement between quintile combinations of Healthy Eating Food Index (HEFI)-2019.and other dietary index systems. 8

7 Supplementary Figure 1. Bland-Altman plots of Canadian Front-of-Pack Labelling (CAN-FOPL) dietary index system compared with other dietary index systems. 9

8 Supplementary Figure 2. Bland-Altman plots of other dietary index systems compared to the Dietary Approaches to Stop Hypertension (DASH) dietary index system. 10

9 Supplementary Figure 3. Bland-Altman plots of dietary index systems compared to the reference standard, Healthy Eating Food Index (HEFI)-2019. 11

10 References 12

1 Supplemental Methods

Five dietary index systems were used in the analysis: Canadian Front-of-Pack Labelling (CAN-FOPL), Diabetes Canada Clinical Practice Guidelines (DCCP), Nutri-score, Dietary Approaches to Stop Hypertension (DASH), and Healthy Eating Food Index (HEFI)-2019.

1.1 Diabetes Canada Clinical Practice Guidelines Dietary Index System

The DCCP nutrient profiling model was used to create the DCCP dietary index system using a 2-step approach (1). The DCCP nutrient profiling model assesses foods and beverages for their alignment with the DCCP through 3 and 2 steps, respectively, allocating 0-2 points at each step (2). Foods are assigned points for their macronutrient or meal quality, saturated and trans fat content, and added sugar content, while beverages are assigned points based on the beverage type, saturated fat, and added sugar content. Points from each step are added, and then converted, when necessary, into a scale of 0 to 6. The final point is used to classify foods and beverages into one of 3 categories for their alignment with the DCCP Guidelines: ‘Least aligned (0-2 points)’, ‘Partially aligned (2.5-4 points)’, and ‘Most aligned (4.5-6 points).’ A higher point indicates better alignment with the DCCP Guidelines (i.e., ‘most healthy’). To assign a dietary index score, first, the raw points from the DCCP nutrient profiling model were standardized on a scale of 0 (‘least healthy’) to 100 (‘most healthy’) (*Equation 1*). Second, the standardized DCCP points were pooled for each participant, weighted by the proportion of energy contributed by each food to get an individual dietary index score (*Equation 2*).

$$\boldsymbol{Standardized\ DCCP\ point} = \frac{Raw\ DCCP\ food/beverage\ point}{6} \times 100 \qquad (\textit{Equation 1})$$

$$\boldsymbol{DCCP\ dietary\ index\ score} = \frac{\sum_{i=1}^{n}(Standardized\ DCCP\ point i) \times E_i}{\sum_{i=1}^{n} E_i} \qquad (\textit{Equation 2})$$

where Standardized DCCP point$_i$ is the standardized food or beverage DCCP-NP point for each food or beverage consumed, and E_i is the energy intake from that food or beverage.

1.2 Nutri-score Dietary Index System

The Nutri-score nutrient profiling model was used to construct the Nutri-Score dietary index scores using a 2-step approach, as previously reported (2). The Nutri-Score is based on the FSAm/HCSP model to determine the healthfulness of foods, which is described in detail elsewhere (3). Briefly, foods and beverages are scored on a continuous scale from -15 (most healthy) to +40 (least healthy) for their energy and nutrient content (total sugars, saturated fats, and sodium as ‘negative’ nutrients and fiber and protein as ‘positive’ nutrients), and the amount of fruits, vegetables, nuts, and legumes. Based on the final point, foods and beverages are classified into 5 categories: Grade A (‘most healthy’), Grade B, Grade C, Grade D, and Grade E (‘least healthy’). To convert the individual food score into a dietary index score, first, foods and beverages in each Nutri-Score category (i.e., Grade A to E) were given points on a scale of 100 (‘most healthy’) to 0 (‘least healthy’) in 25-point increments in descending order. In other words, foods and beverages assigned to Grade A were given 100 points, 75 points for Grade B, 50 points for Grade C, 25 points for Grade D, and 0 point for Grade E (3). Second, these new standardized Nutri-score points were summed for each participant, weighted by the proportion of energy contributed by each food to get an individual dietary index score (*Equation 3*).

$$\textbf{Nutri} - \textbf{score dietary index score} = \frac{\sum_{i=1}^{n}(Nutri\text{–}score\ point i) \times E_i}{\sum_{i=1}^{n} E_i} \quad (Equation\ 3)$$

where Nutri-score point$_i$ is the assigned point (0-100) based on the Nutri-Score nutrient profiling model categories for each food or beverage consumed, and E_i is the energy intake from that food or beverage.

1.3 Dietary Approaches to Stop Hypertension Dietary Index System

The DASH diet is a dietary pattern developed to lower blood pressure and treat hypertension through nutrition therapy (4). The DASH diet emphasizes high intakes of fruits, vegetables, and low-fat dairy products, moderate intakes of whole grains, poultry, fish, and nuts, and low intakes of red meat, sweets, and sugar-containing beverages. First, the DASH dietary index system developed by Matsunaga et al. (5) was used. The DASH dietary index system assigns points proportionally across 9 dietary components (7 food groups: Fruit, Vegetables, Whole Grains, Dairy Products, Plant Proteins, Animal Proteins, and Added Sugars; and 2 nutrients: Sodium and Saturated Fat) addressed in the DASH dietary pattern. Then calorie-based standards for the maximum scores (10 points) are determined using energy-adjusted serving amounts (servings/1,000 kcal) calculated from daily servings for each of the 7 food group components; and maximum scores are assigned based on the recommended daily intakes for the sodium component, and percent to total energy intake for the saturated fat component. The final score is calculated by summing up the scores from each component, resulting in a range from 0 to 90 with a higher total score denoting better adherence to the DASH diet (5). The DASH dietary index scores from individuals were converted into a 100-score system to standardize the scores to the other dietary index systems used in the analysis.

1.4 Healthy Eating Food Index-2019 Dietary Index System

The HEFI-2019 was developed to assess the alignment of diets with Canada's Food Guide (6). Details are published elsewhere (7, 8). Briefly, HEFI-2019 uses 10 components related to key recommendations in Canada's Food Guide (CFG) to assess the dietary alignment with CFG: Vegetables and fruits, Whole-grain foods, Grain foods ratio, Protein foods, Plant-based protein foods, Beverages, Fatty acids ratio, Saturated fats, Free sugars, and Sodium. Scoring standards and the proportional weight of each component were developed based on CFG. The final HEFI-2019 score is calculated by summing up the scores from each component, resulting in a range from 0 to 80 with a higher total score denoting better adherence to CFG. To standardize the scoring system with other dietary index systems, the final score was converted into a 100-score system.

Supplementary Table 1. Energy and nutrient intakes across quintile groups using the Diabetes Canada Clinical Practice Guidelines (DCCP) dietary index system

	Quintile 1 (“Least Healthy”)	Quintile 2	Quintile 3	Quintile 4	Quintile 5 (“Most Healthy”)	P-trend
n	2,699	2,699	2,699	2,699	2,699	
DCCP Dietary index score*	51.1 [50.7, 51.6]	59.6 [59.5, 59.7]	64.4 [64.3, 64.5]	69.0 [68.9, 69.1]	77.4 [77, 77.8]	
Energy (kcal)	2,446 [2,400, 2,492]	2,501 [2,426, 2,575]	2,399 [2,360, 2,439]	2,367 [2,322, 2,412]	2,238 [2,190, 2,286]	<.0001
Total fat (% to total energy)	34.7 [33.7, 35.7]	35.9 [34.2, 37.6]	34.4 [32.9, 36]	33.3 [32.2, 34.4]	30 [27.9, 32.0]	<.0001
Saturated fat (% to total energy)	12.6 [12.2, 13.1]	12.5 [11.6, 13.5]	11.2 [10.9, 11.5]	10.2 [10, 10.5]	8.1 [7.7, 8.5]	<.0001
Protein (% to total energy)	14.6 [13.8, 15.3]	15.8 [15.3, 16.3]	16.7 [16.2, 17.3]	17.8 [17.3, 18.2]	19.1 [18.5, 19.6]	<.0001
Carbohydrates (% to total energy)	48.2 [46.6, 49.8]	45.8 [43.8, 47.9]	46.4 [44.8, 48.1]	47.0 [46, 48.1]	49.4 [47.2, 51.7]	<.0001
Fiber (g/1000 kcal)	6.9 [6.0, 7.7]	7.9 [7.2, 8.6]	8.9 [8.5, 9.3]	10 [9.7, 10.4]	12.8 [12.4, 13.3]	<.0001
Total sugars (% to total energy)	21.9 [20.1, 23.6]	17.8 [16.4, 19.3]	17.3 [15.5, 19]	16.9 [15.6, 18.2]	17.6 [16.3, 19.0]	<.0001
Free sugars (% to total energy)	15.3 [14.1, 16.4]	9.9 [9.1, 10.8]	8.5 [7.6, 9.5]	7.4 [6.7, 8.1]	5.5 [4.8, 6.2]	<.0001
Calcium density (mg/1000 kcal)	389.6 [375.0, 404.2]	433.5 [413.8, 453.2]	428.1 [412.9, 443.3]	418.5 [404.4, 432.5]	417.1 [391.6, 442.6]	0.0005
Vitamin A density in RAE (µg/1000 kcal)	277.9 [246.5, 309.3]	325.7 [301.9, 349.6]	350.9 [266.9, 434.9]	348.0 [325.8, 370.2]	465.4 [395.9, 534.8]	<.0001
Vitamin C density (mg/1000 kcal)	33.6 [29.7, 37.5]	46.5 [41.5, 51.5]	51.0 [46.0, 56.0]	55.7 [51.7, 59.6]	73.9 [69.1, 78.7]	<.0001
Vitamin D density (µg/1000 kcal)	2.1 [2, 2.3]	2.5 [2.2, 2.3]	2.7 [2.3, 3.2]	2.7 [2.5, 2.9]	2.8 [2.5, 3.0]	<.0001
Sodium density (mg/1000 kcal)	1,507.1 [1,438.9, 1,575.2]	1,511.7 [1,467.6, 1,555.9]	1,467.3 [1,423.7, 1,511.0]	1,435.7 [1,393.1, 1,478.2]	1,291.5 [1,238.5, 1,344.5]	<.0001
Iron density (µg/1000 kcal)	6.1 [5.9, 6.3]	6.5 [6.3, 6.7]	6.7 [6.5, 6.9]	6.8 [6.7, 7.0]	7.1 [6.7, 7.5]	<.0001
Potassium density (mg/1000 kcal)	1,157.9 [1,130.6, 1,185.2]	1,309.2 [1,278.7, 1,339.6]	1,449 [1,405.4, 1,492.6]	1,515.7 [1,482.6, 1,548.8]	1,779.2 [1,737.8, 1,820.7]	<.0001

n=13,495; values represent means [95% CI]. Estimates are weighted least squares means from a regression model adjusted for age, sex, misreporting status (under-reporters, plausible reporters and over-reporters), and energy with bootstrapping. P-trends were estimated in their continuous form and represent the P-value associated with the linear regression coefficient. P-trend <0.0001 was considered significant. *DCCP dietary index scores were calculated by assigning points to foods and beverages categorized using the DCCP nutrient profiling model, adjusting the points by the proportion of energy contribution from each food and beverage, and then summing up the energy-adjusted points for a final score. The dietary index score was standardized on a scale of 0 (“least healthy”) to 100 (“most healthy”). Abbreviations: DCCP, Diabetes Canada Clinical Practice Guidelines; RAE, Retinol Activity Equivalents.

Supplementary Table 2. Energy and nutrient intakes across quintile groups using the Nutri-score dietary index system

	Quintile 1 (“Least Healthy”)	Quintile 2	Quintile 3	Quintile 4	Quintile 5 (“Most Healthy”)	P-trend
n	2,699	2,699	2,699	2,699	2,699	
Nutri-score Dietary index score*	36.9 [36.2, 37.6]	47.5 [47.4, 47.7]	54.5 [54.2, 54.7]	62.0 [61.8, 62.2]	74.3 [73.5, 75.1]	
Energy (kcal)	2,493 [2,443, 2,543]	2,494 [2,426, 2,562]	2,432 [2,379, 2,484]	2,299 [2,257, 2,341]	2,207 [2,164, 2,250]	<0.001
Total fat (% to total energy)	37.4 [35.9, 38.9]	35.6 [34.6, 36.5]	34.5 [33.3, 35.6]	31.7 [30.3, 33.1]	28.4 [26.4, 30.4]	<0.001
Saturated fat (% to total energy)	13.3 [12.9, 13.7]	12.2 [11.9, 12.5]	11.1 [10.8, 11.3]	9.8 [9.6, 10.1]	7.9 [7.4, 8.3]	<0.001
Protein (% to total energy)	14.4 [13.7, 15.1]	15.7 [15.2, 16.2]	16.5 [16.2, 16.9]	17.6 [17.1, 18]	20.3 [19.7, 20.9]	<0.001
Carbohydrates (% to total energy)	45.8 [43.8, 47.8]	45.9 [44.4, 47.4]	47.1 [46.1, 48.1]	48.8 [47.1, 50.4]	49.6 [46.7, 52.4]	<0.001
Fiber (g/1000 kcal)	6.5 [6.1, 6.8]	7.9 [7.5, 8.3]	8.9 [8.6, 9.2]	10.5 [10.1, 10.8]	13.3 [12.7, 13.9]	<0.001
Total sugars (% to total energy)	21.4 [19.0, 23.7]	18.9 [17.5, 20.3]	17.6 [16.4, 18.8]	17.1 [15.5, 18.6]	16.0 [14.6, 17.5]	<0.001
Free sugars (% to total energy)	14.3 [12.8, 15.8]	10.8 [10.0, 11.5]	9.1 [8.4, 9.7]	7.6 [6.8, 8.5]	4.9 [4.1, 5.6]	<0.001
Calcium density (mg/1000 kcal)	437.8 [422.3, 453.4]	435.8 [414.6, 457.0]	426.3 [411.1, 441.4]	408.5 [394.3, 422.6]	374.6 [355, 394.2]	<0.001
Vitamin A density in RAE (µg/1000 kcal)	310.6 [288.0, 333.2]	320.7 [299.5, 341.9]	342.0 [316.0, 368.0]	378.1 [310.5, 445.8]	429.9 [386.2, 473.7]	<0.001
Vitamin C density (mg/1000 kcal)	38.3 [34.8, 41.7]	46.9 [36.8, 57.1]	52.5 [46.8, 58.2]	57.5 [52.8, 62.1]	68.3 [62.8, 73.9]	<0.001
Vitamin D density (µg/1000 kcal)	2.5 [2.4, 2.7]	2.6 [2.4, 2.8]	2.5 [2.3, 2.8]	2.4 [2.2, 2.6]	2.7 [2.4, 3.1]	0.10
Sodium density (mg/1000 kcal)	1,472.5 [1,435.9, 1,509.1]	1,505.1 [1,452.9, 1,557.3]	1,443.8 [1,406.8, 1,480.9]	1,441.4 [1,391.8, 1,490.9]	1,335.3 [1,280.5, 1,390.2]	<0.001
Iron density (µg/1000 kcal)	5.8 [5.6, 6.0]	6.3 [6.1, 6.4]	6.7 [6.5, 6.8]	7.1 [6.8, 7.3]	7.5 [7.2, 7.8]	<0.001
Potassium density (mg/1000 kcal)	1,200.9 [1,169.1, 1,232.7]	1,334 [1,295.7, 1,372.4]	1,431.4 [1,368.8, 1,494]	1,518.8 [1,475.3, 1,562.2]	1,780.9 [1,730.6, 1,831.2]	<0.001

n=13,495; values represent means [95% CI]. Estimates are weighted least squares means from a regression model adjusted for age, sex, misreporting status (under-reporters, plausible reporters and over-reporters), and energy with bootstrapping. P-trends were estimated in their continuous form and represent the P-value associated with the linear regression coefficient. P-trend <0.0001 was considered significant. *Nutri-score dietary index scores were calculated by assigning points to foods and beverages categorized using the Nutri-score nutrient profiling model, adjusting the points by the proportion of energy contribution from each food and beverage, and then summing up the energy-adjusted points for a final score. The dietary index score was standardized on a scale of 0 (“least healthy”) to 100 (“most healthy”). Abbreviation: RAE, Retinol Activity Equivalents.

4 **Supplementary Table 3.** Agreement between quintile combinations of the Dietary Approaches to Stop Hypertension (DASH).and other dietary index systems.

		DASH					Discordant pairs*, n (%)	Weighted κ† [95% CI]
		Q1	Q2	Q3	Q4	Q5		
**DCCP**	Q1	19.2	0.7	0.1	0.03	0.02	10,540 (78.1%)	0.07 [0.07, 0.08]
	Q2	18.5	1.2	0.2	0.08	0.02		
	Q3	17.2	2.0	0.5	0.2	0.05		
	Q4	15.0	3.3	1.2	0.4	0.1		
	Q5	11.5	4.3	2.2	1.4	0.6		
**Nutri-score**	Q1	19.2	0.7	0.1	0.01	0	10,459 (77.5%)	0.07 [0.07, 0.07]
	Q2	18.1	1.4	0.3	0.1	0.04		
	Q3	16.4	2.5	0.8	0.3	0.07		
	Q4	14.7	3.3	1.3	0.6	0.2		
	Q5	13.1	3.6	1.7	1.0	0.5		

n=13,495. Increasing quintiles (Q) indicate higher scores (i.e., "healthier" diet quality). Each cell includes the proportion (%) of the total sample falling into the respective quintile combinations. Shaded cells indicate concordant pairs (i.e., samples falling into the same quintile according to the two examined dietary index systems) with 20% in each shaded cell representing perfect agreement, while non-shaded cells indicate discordant pairs (i.e., samples identified as "Less healthy" in one dietary system and "More healthy" in another dietary index system). *Discordant pairs are presented as the total number of identified samples and the proportion (%) of the total sample. †Agreement between dietary index scores were assessed using weighted *κ* statistic, where: 0·01–0·20 represented 'slight' agreement, 0·21–0·40 'fair'; 0·41–0·60 'moderate'; 0·61–0·80 'substantial'; and 0·81–0·99 'near perfect' (9). Abbreviations: DASH, Dietary Approaches to Stop Hypertension Diet; DCCP, Diabetes Canada Clinical Practice Guidelines.

Supplementary Table 4. Agreement between quintile combinations of Healthy Eating Food Index (HEFI)-2019.and other dietary index systems.

		HEFI-2019					Discordant pairs*, n (%)	Weighted κ† [95% CI]
		Q1	Q2	Q3	Q4	Q5		
**DCCP**	Q1	10.7	4.9	2.8	1.2	0.4	8,232 (61.0%)	0.44 [0.43, 0.46]
	Q2	5.2	6.1	4.6	2.9	1.2		
	Q3	2.6	4.8	5.4	4.6	2.6		
	Q4	1.2	3.0	4.9	6.0	5.0		
	Q5	0.4	1.1	2.4	5.3	10.8		
**Nutri-score**	Q1	10.1	5.1	2.8	1.6	0.4	8,461 (62.7%)	0.42 [0.42, 0.44]
	Q2	5.4	5.8	4.8	2.9	1.1		
	Q3	2.8	4.7	5.1	4.7	2.7		
	Q4	1.3	3.0	5.0	5.6	5.1		
	Q5	0.4	1.2	2.3	5.3	10.7		

n=13,495. Increasing quintiles (Q) indicate higher scores (i.e., “healthier” diet quality). Each cell includes the proportion (%) of the total sample falling into the respective quintile combinations. Shaded cells indicate concordant pairs (i.e., samples falling into the same quintile according to the two examined dietary index systems) with 20% in each shaded cell representing perfect agreement, while non-shaded cells indicate discordant pairs (i.e., samples identified as “Less healthy” in one dietary system and “More healthy” in another dietary index system). *Discordant pairs are presented as the total number of identified samples and the proportion (%) of the total sample. †Agreement between dietary index scores were assessed using weighted *κ* statistic, where: 0·01–0·20 represented ‘slight’ agreement, 0·21–0·40 ‘fair’; 0·41–0·60 ‘moderate’; 0·61–0·80 ‘substantial’; and 0·81–0·99 ‘near perfect’ (9). Abbreviations: DCCP, Diabetes Canada Clinical Practice Guidelines; HEFI, Healthy Eating Food Index.

Supplementary Figure 1. Bland-Altman plots of Canadian Front-of-Pack Labelling (CAN-FOPL) dietary index system compared with other dietary index systems.

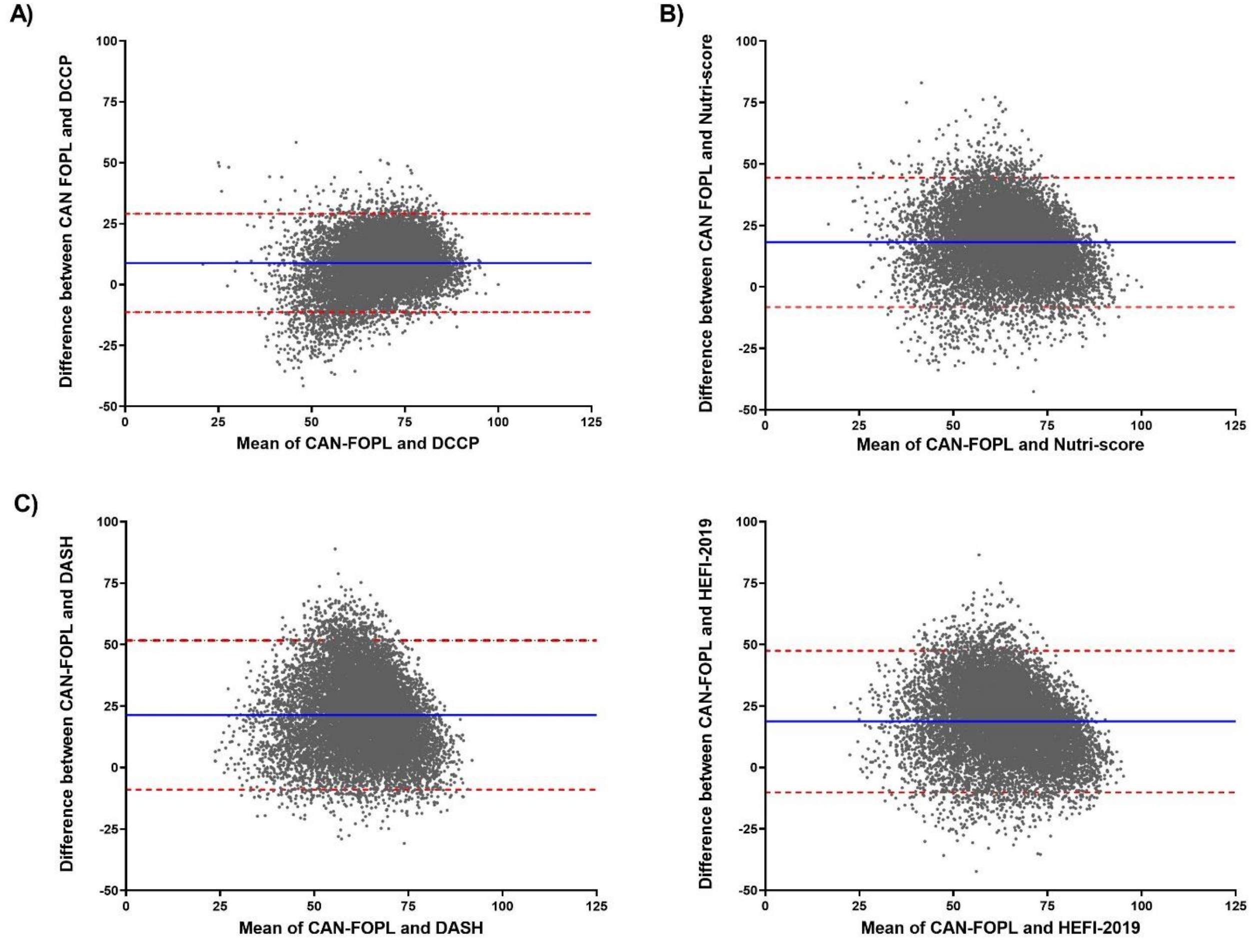

Dietary index scores from (A) DCCP, (B) Nutri-score, (C) DASH, and (D) HEFI-2019 were compared to CAN-FOPL dietary index scores. Solid blue lines represent the mean differences, while the dashed red lines represent 95% limits of agreement (LOA). n=13,495. Mean differences [95% LOA] were (A) 8.8 [-11.3, 29.0]; (B) 18.1 [-8.2, 44.4]; (C) 21.3 [-9.0, 51.7]; and (D) 18.7 [-10.1, 47.5]). Abbreviations: CAN-FOPL, Canadian Front-of-Pack Labelling; DASH, Dietary Approaches to Stop Hypertension Diet; DCCP, Diabetes Canada Clinical Practice Guidelines; HEFI, Healthy Eating Food Index; LOA, Limits of Agreement.

Supplementary Figure 2. Bland-Altman plots of other dietary index systems compared to the Dietary Approaches to Stop Hypertension (DASH) dietary index system.

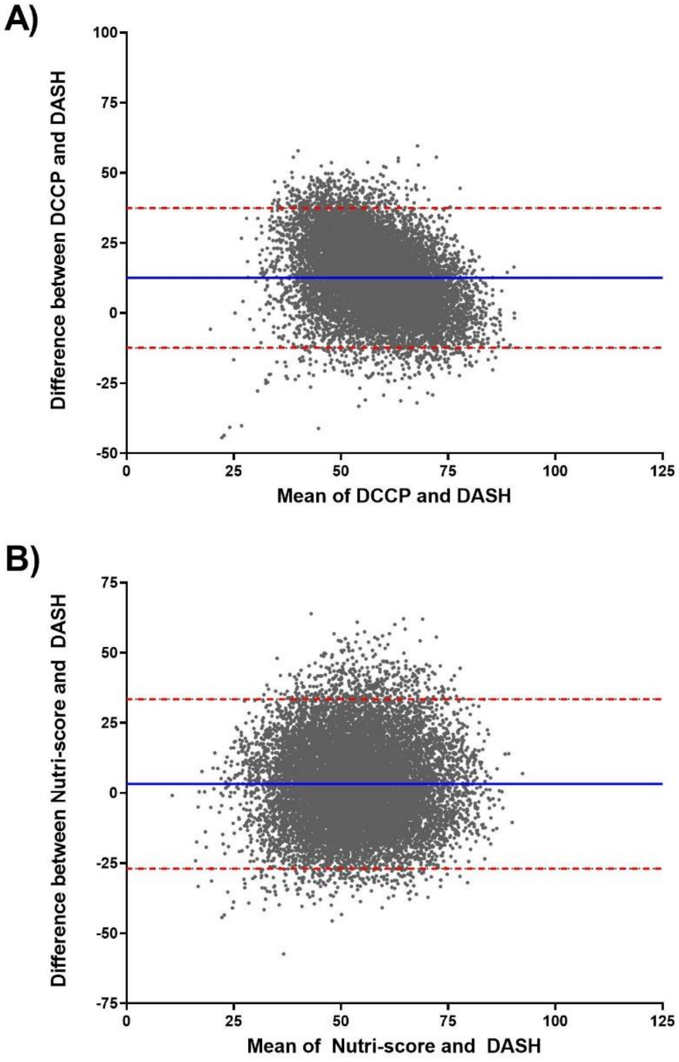

Dietary index scores from (A) DCCP and (B) Nutri-score were compared to DASH. Solid blue lines represent the mean differences, while the dashed red lines represent 95% limits of agreement (LOA). n=13,495. Mean differences [95% LOA] were as follows: (A) 12.5 [-12.4, 37.4] and (B) 3.2 [-27.0, 33.4]. Abbreviations: DASH, Dietary Approaches to Stop Hypertension Diet; LOA, Limits of Agreement.

9 **Supplementary Figure 3.** Bland-Altman plots of dietary index systems compared to the reference standard, Healthy Eating Food Index (HEFI)-2019.

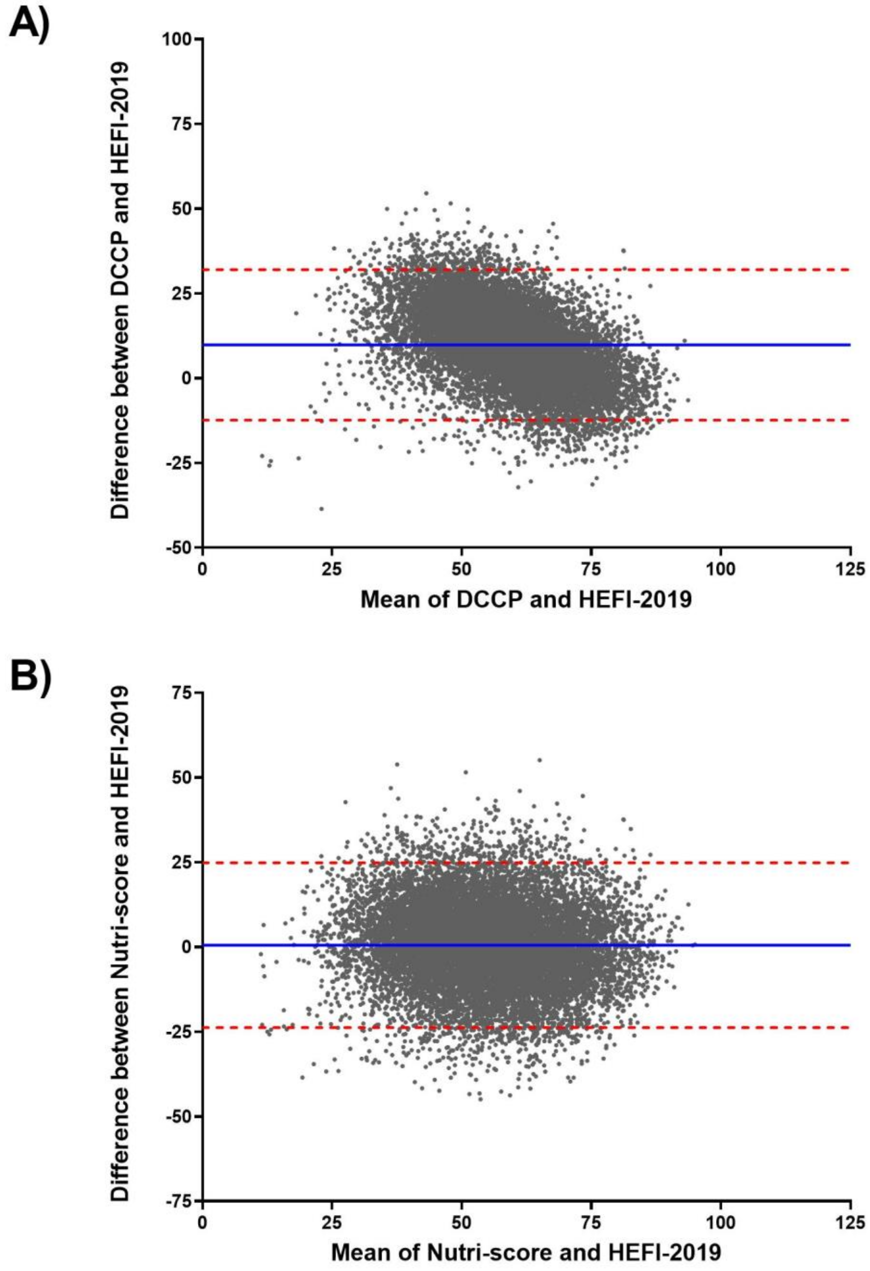

Dietary index scores from (A) DCCP and (B) Nutri-score were compared to HEFI-2019. Solid blue lines represent the mean differences, while the dashed red lines represent 95% limits of agreement (LOA). n=13,495. Mean differences [95% LOA] were as follows: (A) 9.8 [-12.4, 32.0]) and (B) 0.5 [-23.8, 24.8]). Abbreviations: DCCP, Diabetes Canada Clinical Practice Guidelines; HEFI-2019, Healthy Eating Food Index 2019; LOA, Limits of Agreement.

10 References

1. Ahmed M, Weippert, M., Nayed, N., Mulligan, C., Vergeer, N., Franco-Arellano, B., Julia, C., L'Abbe, M. R. Developing a Nutrient Profiling Model to Align with the Healthy Eating Recommendations of the Diabetes Canada Clinical Practice Guidelines [Abstract]. *Appl Physiol Nutr Metab* (2021) 46(4 (Suppl. 1)):S1. doi: 10.1139/apnm-2021-0172.

2. Paper L, Ahmed M, Lee JJ, Kesse-Guyot E, Touvier M, Hercberg S, et al. Cross-Sectional Comparisons of Dietary Indexes Underlying Nutrition Labels: Nutri-Score, Canadian 'High in' Labels and Diabetes Canada Clinical Practices (Dccp). *Eur J Nutr* (2022). doi: 10.1007/s00394-022-02978-w.

3. Santé Publique France. Nutri-Score Scientific and Technical Questions and Answers. Saint-Maurice: Santé publique France.(2018) [accessed July 20, 2021]. Available at: https://www.santepubliquefrance.fr/determinants-de-sante/nutrition-et-activite-physique/articles/nutri-score.

4. Appel LJ, Moore TJ, Obarzanek E, Vollmer WM, Svetkey LP, Sacks FM, et al. A Clinical Trial of the Effects of Dietary Patterns on Blood Pressure. Dash Collaborative Research Group. *N Engl J Med* (1997) 336(16):1117-24. doi: 10.1056/nejm199704173361601.

5. Matsunaga M, Hurwitz EL, Li D. Development and Evaluation of a Dietary Approaches to Stop Hypertension Dietary Index with Calorie-Based Standards in Equivalent Units: A Cross-Sectional Study with 24-Hour Dietary Recalls from Adult Participants in the National Health and Nutrition Examination Survey 2007-2010. *J Acad Nutr Diet* (2018) 118(1):62-73.e4. doi: 10.1016/j.jand.2017.03.010.

6. Health Canada. Canada's Food Guide (2019) [accessed March 7, 2020]. Available at: https://food-guide.canada.ca/en/.

7. Brassard D, Munene L-AE, St-Pierre S, Guenther PM, Kirkpatrick SI, Slater J, et al. Development of the Healthy Eating Food Index (Hefi)-2019 Measuring Adherence to Canada's Food Guide 2019 Recommendations on Healthy Food Choices. *Physiol Nutr Metab* (2022) 47(5):595-610. doi: 10.1139/apnm-2021-0415.

8. Brassard D, Munene L-AE, St-Pierre S, Gonzalez A, Guenther PM, Jessri M, et al. Evaluation of the Healthy Eating Food Index (Hefi)-2019 Measuring Adherence to Canada's Food Guide 2019 Recommendations on Healthy Food Choices. *Appl Physiol Nutr Metab* (2022) 47(5):582-94. doi: 10.1139/apnm-2021-041.

9. Viera AJ, Garrett JM. Understanding Interobserver Agreement: The Kappa Statistic. *Fam med* (2005) 37(5):360-3.
